# Long-term survival in thymic carcinoma with postoperative pleural dissemination

**DOI:** 10.1186/s40792-021-01255-y

**Published:** 2021-07-30

**Authors:** Toru Kimura, Masahiko Higashiyama, Keiichiro Honma, Harumi Nakamura, Tomohiro Maniwa, Jiro Okami

**Affiliations:** 1 Department of General Thoracic Surgery, Osaka International Cancer Institute, 1,3,4: 3-1-69, Otemae, Chuo-ku, Osaka 541-8567 Japan; 2 Department of General Thoracic Surgery, Higashiosaka City Medical Center, 2: 3-4-5, Nishiiwata, Higashiosaka, Osaka 578-8588 Japan; 3 Department of Pathology, Osaka International Cancer Institute, 1,3,4: 3-1-69, Otemae, Chuo-ku, Osaka 541-8567 Japan; 4 Laboratory of Genomic Pathology, Osaka International Cancer Institute, 1,3,4: 3-1-69, Otemae, Chuo-ku, Osaka 541-8567 Japan

**Keywords:** Thymic carcinoma, Postoperative recurrence, Disseminated pleural nodules, Long-term survival

## Abstract

**Background:**

We report a patient with thymic squamous cell carcinoma who underwent multiple rounds of surgical resection and definitive radiotherapy for both primary tumor and postoperative recurrence. However, the patient remains well and healthy 18 years after initial diagnosis. Since long-term survival after postoperative recurrence of thymic carcinoma is extremely rare, we also present her immunohistochemical staining results, which suggested indolent disease.

**Case presentation:**

A 42-year-old woman with thymic squamous cell carcinoma underwent en bloc resection of the tumor and thymus gland. Pleural dissemination was noted in the right thoracic cavity 3, 10, and 16 years postoperatively. Where possible, the nodules were resected surgically: during the postoperative 3rd and 16th years. Definitive radiotherapy was administered for all nodules that could not be excised during the postoperative 3rd and 10th years. Disease-free survival is 25 months.

**Conclusions:**

Local control of pleural dissemination may be beneficial in the treatment of postoperative recurrence of thymic carcinoma in limited cases of indolent disease.

## Background

Thymic carcinoma is a rare and highly aggressive mediastinal neoplasm. Unlike in thymoma, the benefits of surgery and radiotherapy (RT) for pleural dissemination of thymic carcinoma remain unknown. Herein, we report an unusual case of long-term survival following multiple rounds of surgical resection and definitive RT for disseminated pleural nodules in a patient with thymic carcinoma.

## Case presentation

**Fig. 1:**
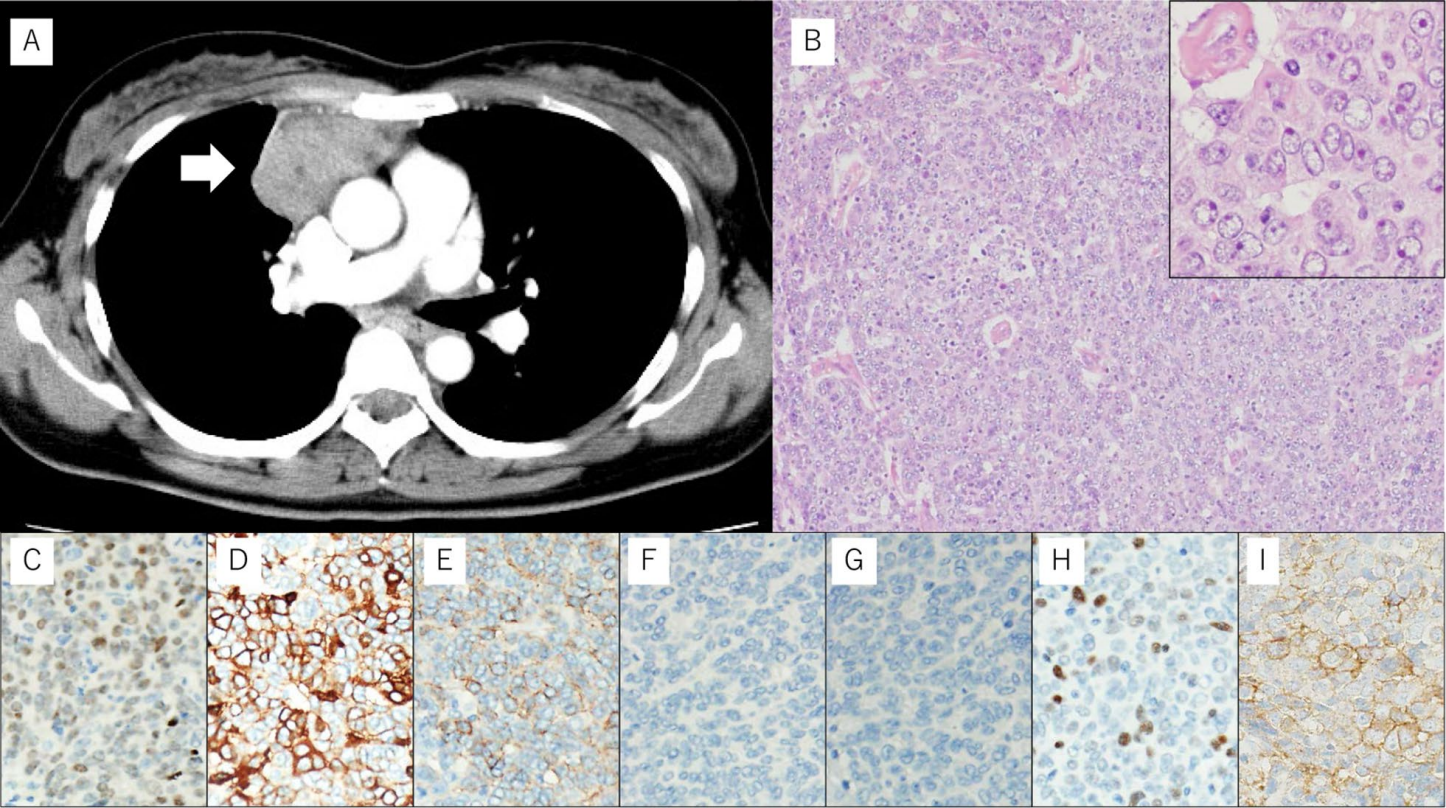
**A** Chest computed tomography imaging of the primary tumor (arrow). **B** Pathological examination of the primary tumor reveals thymic squamous cell carcinoma. Hematoxylin and eosin staining; original magnification ×50 and ×200 (inset). **C**–**I** On immunohistochemical staining, the primary tumor is positive for *PAX8* (**C**), CD5 (**D**), and *c-KIT* (**E**), while intratumoral lymphocytes are negative for TdT (**F**) and CD1a (**G**). The primary tumor demonstrates low expression of p53 (**H**) and positive staining for programmed death-ligand 1 (**I**). Original magnification ×200

**Fig. 2:**
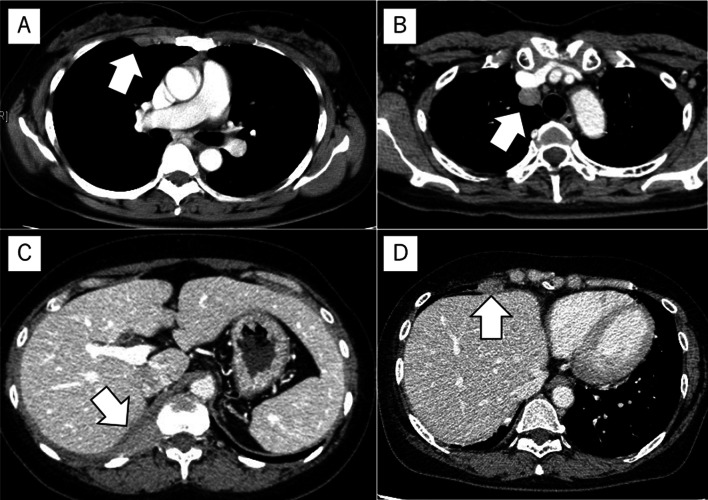
Chest computed tomography (CT) imaging of the disseminated pleural nodules seen in the postoperative recurrence of thymic carcinoma (arrows). **A** CT taken during the 3rd postoperative year. A nodule is noted on the anterior chest wall. **B**, **C** CT taken during the 10th postoperative year. A nodule is observed right behind the right brachiocephalic vein (**B**) and at the crus of the diaphragm (**C**). **D** CT taken during the 16th postoperative year. A nodule is shown between the anterior chest wall and the diaphragm

**Fig. 3:**
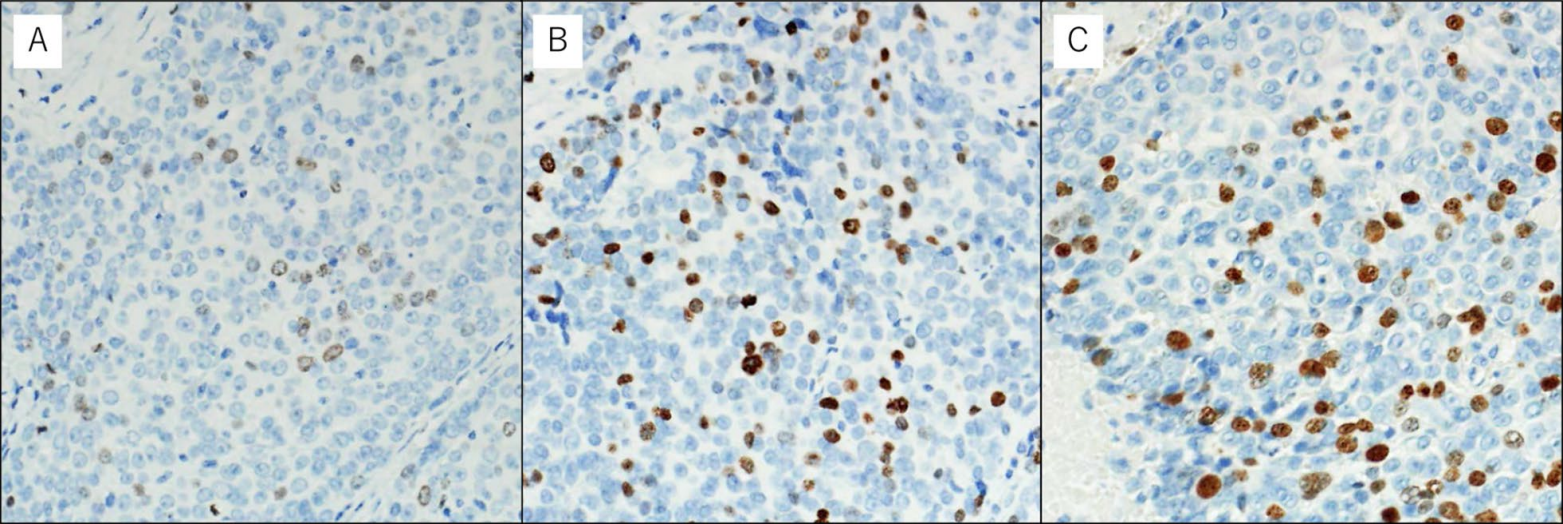
Immunohistochemical staining of the primary tumor and disseminated nodules for Ki-67. The fraction of Ki-67-positive tumor cells in the primary tumor (**A**), disseminated nodule at the third postoperative year (**B**), and that at the 16th postoperative year (**C**) were 10%, 15%, and 25%, respectively. Original magnification ×200

A 42-year-old woman presented with a mediastinal tumor and anterior chest pain. Computed tomography (CT) revealed a right anterior mediastinal tumor measuring 7.0 $\times$ 4.2 $\times$ 3.2 cm (Fig. 1A). CT-guided core-needle biopsy was performed. Since the tumor had been diagnosed as thymoma and estimated to be resectable at initial surgery, she underwent en bloc resection of the tumor and entire thymus gland through a full median sternotomy. Pathological examination revealed that the tumor was moderately differentiated thymic squamous cell carcinoma (SCC), which showed atypical cells varying in size and shape and proliferating in a sheet-like pattern (Fig. 1B). Immunohistochemical staining for *PAX8,* CD5, *c-KIT*, TdT, and CD1a also demonstrated features that are characteristic to thymic carcinoma (Fig. 1C–G). The tumor was graded as pT2 N0 M0, p-Stage II according to the Union for International Cancer Control TNM eighth edition classification. During the third postoperative year, a follow-up CT demonstrated multiple nodules in the right thoracic cavity, which were characteristics of pleural dissemination. We resected all visible nodules on the visceral pleura except for a nodule with firm adhesions located on the anterior chest wall (Fig. 2A). We administered four courses of systemic chemotherapy with carboplatin and etoposide and delivered definitive RT at a dose of 60 Gy directly to the nodule. The patient developed rheumatic arthritis (RA) over the course of this treatment. During the tenth postoperative year, a follow-up CT revealed two nodules in the right thoracic cavity (Fig. 2B, C). We selected RT over surgical resection due to previous intraoperative findings in this patient. RT at a dose of 60 Gy was delivered to each of the nodules. During the 16th postoperative year, a follow-up CT revealed another pleural nodule (Fig. 2D), which was resected through a horizontal incision made just above the lesion. Histopathological examination of the resected pleural nodule confirmed establishing a similar diagnosis of thymic squamous cell carcinoma. Immunohistochemical staining for Ki-67 revealed that proliferation of tumor cells gradually increased (Fig. 3A–C). Immunohistochemical staining for p53 and programmed death-ligand 1 (PD-L1 using 22C3 anti-PD-L1 antibody) in the primary tumor (Fig. 1H, I) and this nodule demonstrated that both were positive for wild-type *TP53* and PD-L1. Twenty-five months following the last surgery, the patient remained in good health. No new lesions were noted. Her RA is well controlled with medication.

## Discussion

Studies of thymic carcinoma have consistently demonstrated a significant survival advantage in surgically treated patients (1). Multidisciplinary treatment with surgical resection, chemotherapy, and RT improves prognosis even in cases of advanced disease or incomplete resection [1, 2]. Comparatively, prognosis remains poor in patients with postoperative recurrence of thymic carcinoma [3]. In a report by Hamaji et al*.* [3] in which they evaluated nine patients with recurrent thymic carcinoma, none survived more than 5 years.

Unlike thymomas, thymic carcinomas are infrequently associated with autoimmune disorders, such as myasthenia gravis and RA [4]. The clinical course of metachronous disseminated pleural nodules coupled with a long-term survival rate in our patient is also more peculiar to thymoma rather than to thymic carcinoma [3, 4]. However, immunohistochemical evaluation, including expression patterns of *PAX8,* CD5, *c-KIT*, TdT, and CD1a that are characteristic to thymic carcinoma [5], confirmed the diagnosis in our patient. None of the recurrent pleural nodules were shown to have originated from a thymoma or any other organ. Next-generation sequencing has demonstrated that *TP53* is the most frequently mutated gene in thymic cancer. Alterations in p53 have also been shown to be associated with a worse prognosis [6]. The present case demonstrated low-level expression of p53 in less than 50% of tumor cells [6], which may be compatible with a favorable prognosis in our patient. While PD-L1 positivity in thymic carcinomas has been reportedly shown to be 41%–100% [7], there is no consensus on whether high PD-L1 expression in thymic carcinoma is associated with better [8] or worse [9] outcomes. T cells expressing the programmed death-1 (PD-1) co-receptor have also been correlated with the pathogenesis of RA [10]. However, correlation between PD-L1 expression on thymic epithelial tumors and RA as a comorbidity is still not well understood. Additional research on PD-L1 expression and its biological significance in thymic carcinoma is needed due to the rarity of this disease.

Surgical resection has been shown to result in favorable long-term outcomes in patients with disseminated pleural nodules from both thymoma [11] and postoperative recurrence of thymic carcinoma [3, 12]. Prognosis of thymic carcinoma is further improved with neoadjuvant and adjuvant RT, as well as concurrent or sequential RT and chemotherapy [13]. Successful RT as part of the multidisciplinary treatment of thymic carcinoma suggests that thymic carcinoma may have cellular sensitivity to RT. However, only a limited number of case reports have documented this success to date [14, 15]. Our findings suggest that surgical resection and definitive RT of pleural nodules may contribute to better outcomes in selected cases of thymic carcinoma.

## Conclusion

We encountered a rare case of long-term survival in a patient who underwent multiple rounds of surgical resection and RT for primary thymic carcinoma and its subsequent recurrences. Local control of pleural dissemination may be beneficial for the treatment of postoperative recurrent thymic carcinoma in limited cases with indolent disease.

## Data Availability

Data sharing is not applicable to this article as no datasets were generated or analyzed during the current study.

## Abbreviations

CT: Computed tomography
PD-L1: Programmed death-ligand 1
RA: Rheumatic arthritis
RT: Radiotherapy
